# Assessing women’s empowerment, participation, and engagement in aquaculture in Bangladesh

**DOI:** 10.1007/s10499-024-01467-7

**Published:** 2024-04-11

**Authors:** Lucy Njogu, Rahma Adam, Cathy Rozel Farnworth

**Affiliations:** 1 WorldFish Kenya, c/o International Livestock Research Institute, Nairobi, 00100 Kenya; 2 School of Global Development, University of East Anglia, Norwich, UK; 3 Pandia Consulting, Teigelkamp 64, 48145 Münster, Germany

**Keywords:** Women’s empowerment, Gender, Aquaculture, Fish, Bangladesh

## Abstract

Women’s empowerment and gender equality are key goals for development and human rights. However, a significant gap still exists in achieving these twin goals. Formulating appropriate strategies for women’s empowerment requires first understanding context-specific patterns and sources of disempowerment. We use data collected using a questionnaire survey from 1653 households in Rangpur and Rajshahi districts in Bangladesh. Guided by an analytic tool that measures women’s empowerment, inclusion and agency (the project level Women’s Empowerment in Fisheries and aquaculture Index (pro-WEFI)), and using seven empowerment indicators, we provide findings on the status of women’s empowerment, participation, and engagement in aquaculture in Bangladesh. Results show that women were highly involved in making household decisions, mainly jointly with their husbands. However, data suggest a substantial gap in women’s access to financial services, in participation in aquaculture activities, and in access to and control over productive capital and remuneration for aquaculture labor. Finally, despite some women achieving adequacy on some indicators, most women in fish farming households in Bangladesh lack adequacy on many of the selected indicators.

## 1. Introduction

Worldwide, aquaculture supports livelihoods based on the sustainability of life below water (Sustainable Development Goal - SDG 14). Aquaculture has the potential to play a significant role in reducing hunger (SDG 2), eliminating poverty (SDG 1), and gender equality (SDG 5) (Troell et al. 2023). Bangladesh is the fifth-largest aquaculture fish producer worldwide (FAO 2017; Murphy et al. 2020). An estimated 4.27 million Bangladeshi households have at least one fishpond (Choudhury et al. 2017). In rural Bangladesh, fish play an important role in income generation and household food consumption. It provides between 2.8 and 15% of household income, and 26–47% of household food consumption (Choudhury et al. 2017). Fish contribute about 60% of animal protein intake in poor rural households (Shamsuzzaman et al. 2020). On average, 13% of household expenditure is on fish (Apu et al. 2014). However, the subsector is yet to achieve its full potential (Islam et al. 2016; Jui and Rahman 2018). Productivity is low (Kruijssen et al. 2016. More rapid aquaculture development is necessary to meet increasing fish demand, given that per capita consumption is expected to rise (Haque et al. 2020). Ensuring that this development is gender-equitable is an important challenge facing development actors.

Women are important in aquaculture in Bangladesh, though comprehensive data about the importance of women in aquaculture is lacking (Rahman and Naoroze 2007; Huq et al. 2016). Involving women in paid aquaculture work promotes their purchasing power and financial freedom (FAO 2017). It also increases their social status, family income, aquaculture productivity, and nutrition (Farnworth et al. 2015; Jui and Rahman 2018; Kruijssen et al. 2018). Reports based on national datasets indicate that Bangladesh has made significant strides towards gender parity in a range of sectors through increasing the participation of women in a variety of political and socio-economic activities (Center or Research and Information 2019). However, the limited data available suggests high levels of gender-based inequality in aquaculture (Ishita 2019; Huq et al. 2016; Choudhury and McDougall 2018). There is still a dearth of information on women’s involvement in aquaculture in Bangladesh and the degree to which their participation has the potential to be empowering (Aregu et al. 2018; Kruijssen et al. 2018).

This study aims to contribute to the limited database. It aims to describe the existing characteristics of women in aquaculture households and it assesses women’s empowerment using a range of indicators. To do this, it analyses data drawn from a survey applied in 2020. This survey drew upon the pro-Women’s Empowerment in Fisheries Index (pro-WEFI) for its structure and analytic categories. Although the survey does not represent an application of the pro-WEFI, it does allow similar comparative data to be constructed and analyzed.

## 2. Methodology

### 2.1 Data collection

Data used in this study were drawn from a large dataset created by the Aquaculture: Increasing income, diversifying diets, and empowering women (IDEA) in Bangladesh project baseline study (Worldfish 2020) which was funded by Bill & Melinda Gates Foundation (BMGF). The project focused on exploiting the untapped aquaculture potential in Bangladesh (Barooah et al. 2022), particularly in Rangpur and Rajshahi divisions. High rates of undernutrition and poverty levels, especially among women and children, are reported in this area (Karim et al. 2021). Rajshahi and Rangpur are neighbor divisions located in the Northwestern region of Bangladesh, home to close 34 million people (Haque et al. 2020). The two divisions are served by the Ganga, Jamuna, and Brahmaputra rivers. These rivers provide conducive agro-ecologies for fish farming (Haque et al. 2020). Rajshahi division is located between 23°48′ and 25°16′ north latitudes and 88°01′ and 89°48′ east longitudes and measures 17974.68 sq km, with an average population of 20 million people (Barooah et al. 2022; Karim et al. 2021). Rangpur Division is located between 25°20′ and 26°37′ north latitudes and 88°50′ and 89°53′ east longitudes, measures about 16,185 square kms, with an average a population of about 17 million people (Barooah et al. 2022).

**Fig. 1:**
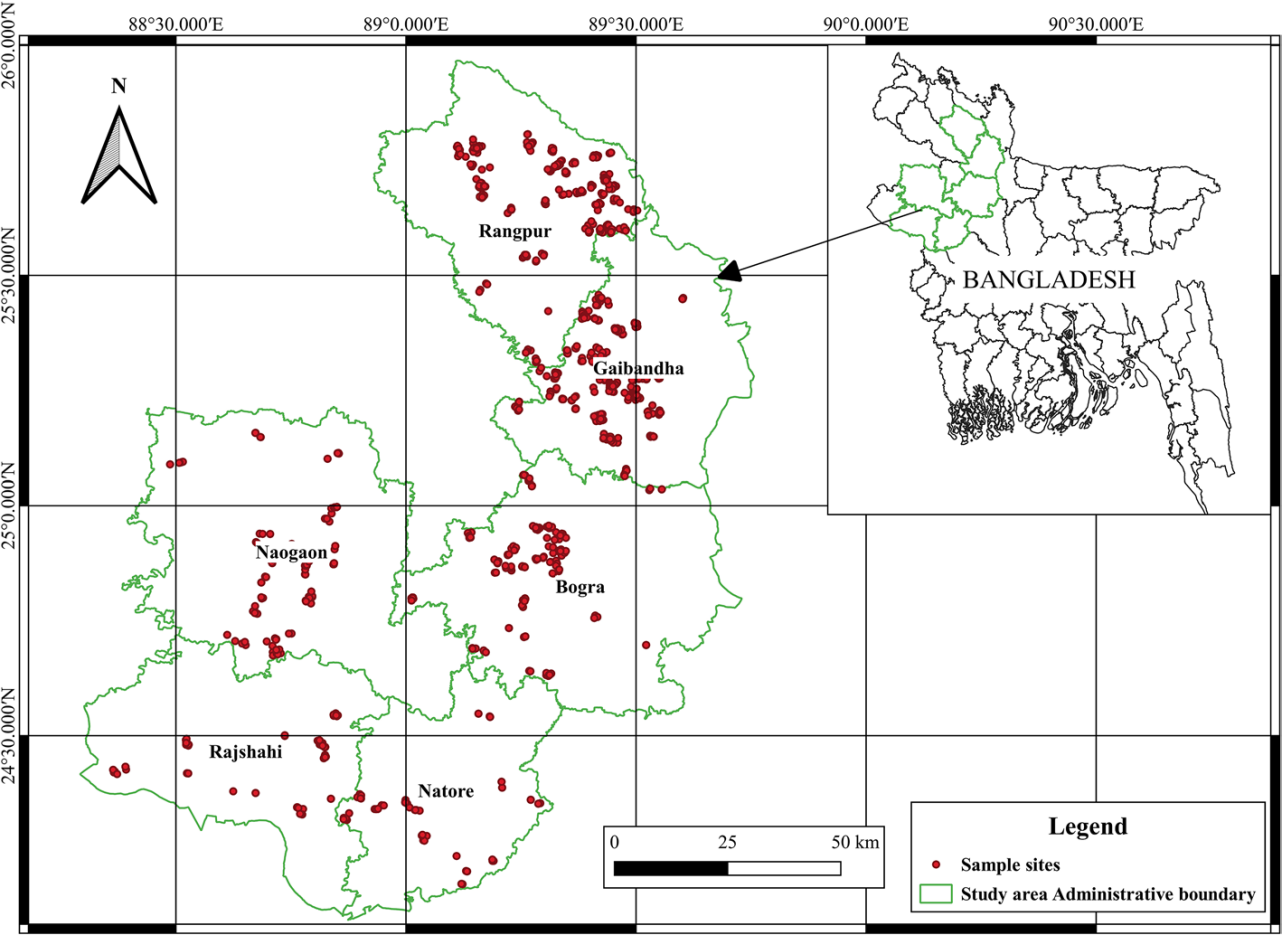
Selected research areas in Rangpur and Rajshahi divisions in northwestern Bangladesh

The IDEA project used a household survey questionnaire to obtain data in relation to indicators for women’s empowerment from aquaculture households. The survey questionnaire was administered to the primary decision-making woman in 1653 households from Rangpur and Rajshahi divisions (see Fig. 1), while data on household sharing of aquaculture activities, hiring, and compensation for fish farming labor was collected from the household head. The survey was conducted in 2020 and the reference period was from June/July 2019 to June/July 2020 (reflecting the aquaculture production year). A stratified random sampling procedure was adopted for sample selection. The first level of categorization was based on business models (practice and outreach group and hybrid model). The second level of categorization was made based on the type of engagement of the farmers with the local service providers (LSPs) (practice group and outreach). Finally, the sample size for each group was determined based on 95% confidence and 5% precision. A total of 878 households in Rajshahi division and 775 households in Rangpur divisions were interviewed. In Rajshahi division, data was collected in Bogura, Naogan, Natore, and Rajshahi districts while in Rangpur, data was collected from Gaibandha and Rangpur districts. In total, data were collected from 36 Upazilas and 344 villages.

### 2.2 Data and concepts used for analysis

The author team decided to use the pro-WEFI analytic framework to assess the IDEA survey questionnaire data. Pro-WEFI is a development of the Pro-Women’s Empowerment in Agriculture Index (pro-WEAI), which in turn draws upon the Women’s Empowerment in Agriculture Index (WEAI). The WEAI is a survey-based index which directly measures women’s empowerment, agency, and inclusion in the agricultural sector at country or regional level (Malapit et al. 2014, 2015). Since its creation in 2012, WEAI has undergone several adaptations. Pro-WEFI was tailored for projects in aquaculture and fisheries to facilitate the standardized measurement of the empowerment, agency, and inclusion of women.

Pro-WEFI is a composite of women’s empowerment and gender parity sub-indices. The women’s empowerment sub-index is weighted at 90% for pro-WEFI and the gender parity sub-index at 10%. The empowerment sub-index reflects the degree of women’s empowerment based on three domains of empowerment (3DE) while the gender parity index (GPI) shows the proportion of women who are as empowered as the men in their households. For those households that have not achieved gender parity, the GPI sub-index shows the gap that needs to be closed for women to reach the same level of empowerment as men. In this study, only women were interviewed and so the GPI sub-index was not calculated.

**Fig. 2:**
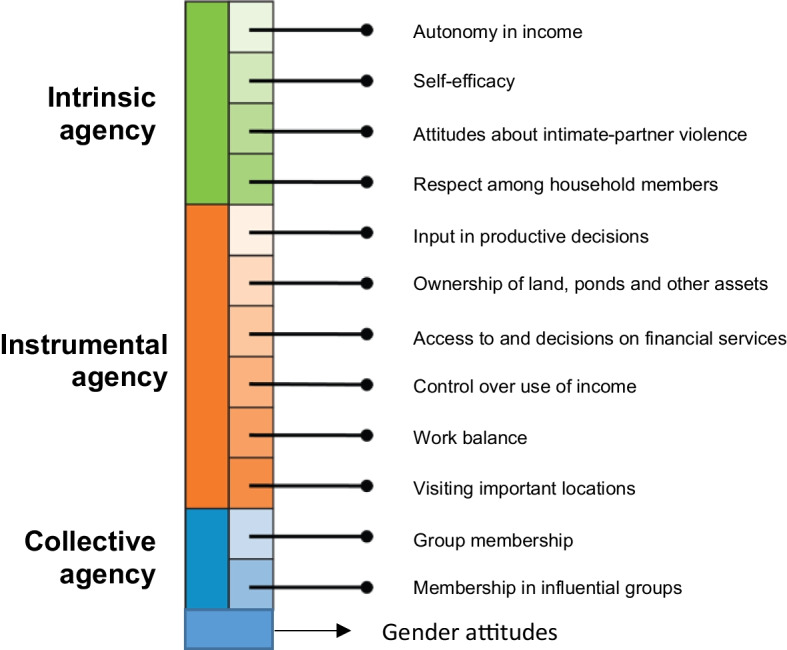
The domains for pro-WEFI (Source: McDougall et al. 2022)

The 3DE is comprised of intrinsic agency (power within), instrumental agency (power to), and collective agency (power with) (Malapit et al. 2019; Quisumbing et al. 2021b; Yount et al. 2019). It has 12 indicators (see Fig. 2). The gender attitude indicator was added to the pro-WEFI to help measure a project’s gender-transformative impacts (Quisumbing et al. 2021a). Pro-WEFI data are collected through a 3DE survey administered to men and women from the same households.

An individual is scored as adequate or inadequate for each of the 12 indicators, which are equally weighted (Table 1). Each indicator has a pre-determined adequacy threshold. For instance, a woman is considered to have adequacy in the control over use of income if she has input in decisions on how to use income and output from all household agricultural and non-agricultural activities (provided a decision was actually made). A score of “1” is allocated to indicate adequacy, or “0” to indicate that the adequacy threshold was not met. The empowerment score is arrived at by aggregating the scores from the 12 indicators. If the respondent has an adequacy score of 9 out of 12 or above, he/she is considered empowered (Malapit et al. 2019). Conversely, respondents who do not achieve adequacy in 25% or more (4 out of 12 indicators) are categorized as disempowered.

**Table 1:** Adequacy threshold for pro-WEFI and adaptations made in the current study

	Pro-WEFI	Current study
Intrinsic agency		
Autonomy in income	More motivated by own values than by coercion or fear of others’ disapproval: relative autonomy index (RAI) B score ≥ 1. RAI score is calculated by summing responses to the three vignettes about a person’s motivation for how they use income generated from agricultural and non-agricultural activities (yes = 1; no = 0), using the following weighting scheme: 0 for vignette 1 (no alternative), 2 for vignette 2 (external motivation), 1 for vignette 3 (introjected motivation), and + 3 for vignette 4 (autonomous motivation)	Involved in any income-generating activities independently and takes part in making decisions on how the income was to be used, either individually or jointly with spouses or other household members.
Self-efficacy	“Agree” or greater on average for self-efficacy questions: new general self-efficacy scale C score ≥ 32	
Attitudes about domestic violence	Believes husband is NOT justified in hitting or beating his wife in all 5 scenarios: (1) She goes out without telling him, (2) She neglects the children, (3) She argues with him, (4) She refuses to have sex with him, (5) She burns the food	
Intrahousehold relationships	Meets ALL the following conditions related to their spouse, the other respondent, or another household member: (1) Respondent respects relation (MOST of the time) AND, (2) Relation respects respondent (MOST of the time) AND, (3) Respondent trusts relation (MOST of the time) AND, (4) Respondent is comfortable disagreeing with relations (MOST of the time)	To assess adequacy in intrahousehold relations 4 statements were posed (Table 2). Women who responded, “high extent” or “medium extent” to each the four statements were allocated a score of “1,” implying that they achieved adequacy in this indicator.
Instrumental agency		
Input in productive decisions	Meets at least ONE of the following conditions for ALL of the agricultural activities they participate in (1) Makes related decision solely, (2) Makes the decision jointly and has at least some input into the decisions, (3) Feels could make decision if wanted to (to at least a MEDIUM extent)	Results were presented in descriptive statistics (percentages) (Tables 2 and 4)
Access to and control over productive capital	Owns, either solely or jointly, at least ONE of the following: (1) At least THREE small assets (poultry, non-mechanized equipment, or small consumer durables), (2) At least TWO large assets, (3) Land	Women whose households had at least one of the two assets and had their names in the ownership documents, either solely or jointly with other people, were considered to have achieved adequacy in this indicator
Access to financial services	Meets at least ONE of the following conditions: (1) Belongs to a household that used a source of credit in the past year AND participated in at least ONE sole or joint decision about it, (2) Belongs to a household that did not use credit in the past year but could have if it wanted to from at least ONE source, (3) Has access, solely or jointly, to a financial account	Respondent’s households had used credit either in cash or kind, in the past one year, and respondent had participated in deciding to take loan and how the loans were to be used. Also, respondent has a financial account, (digital or at any financial institution), and makes decisions about utilizing the account and ownership of the account (Table 2).
Control over use of income	Has input in decisions related to how to use BOTH income and output from ALL of the agricultural activities they participate in AND has input in decisions related to income from ALL non-agricultural activities they participate in, unless no decision was made	Respondents were asked about their involvement in making decisions regarding saving money, investing in income-generating activities, and routine household purchases of basic items such as food for daily consumption or other household needs, where the household made such decisions. Respondents who had participated in making the decisions either solely or jointly with other people were considered to have achieved adequacy in this indictor.
Time use agency	Works less than 10.5 h per day: workload = time spent in primary activity + (1/2) time spent in childcare as a secondary activity	
Physical mobility	Meets at least ONE of the following conditions: (1) Visits at least TWO locations at least ONCE PER WEEK of (city, market, family/relative) or (2) Visits at least ONE location at least ONCE PER MONTH of (health facility, public meeting)	
Collective agency		
Group membership	Active member of at least ONE group	Being member of a group
Membership in influential groups	Active member of at least ONE group that can influence the community to at least a MEDIUM extent	

In this study, data were collected on seven indicators from female respondents. The authors computed indicators for autonomy in income, intrahousehold relationships, access to and control over productive capital, control over income, financial empowerment, and group membership. Results on input in productive and income decisions were analyzed through descriptive statistics, and not as a final indicator as generated in pro-WEFI. We also analyzed the gender attitude which is not a main component of the pro-WEFI, but usually added as a separate indicator in the WEAI and WEFI tools. It is intended to indicate the state of gender attitudes before and after intervention. The results on autonomy in income and input in income and productive decisions were presented using descriptive statistics.

## 3.Results

### 3.1 Descriptive statistics

**Fig. 3:**
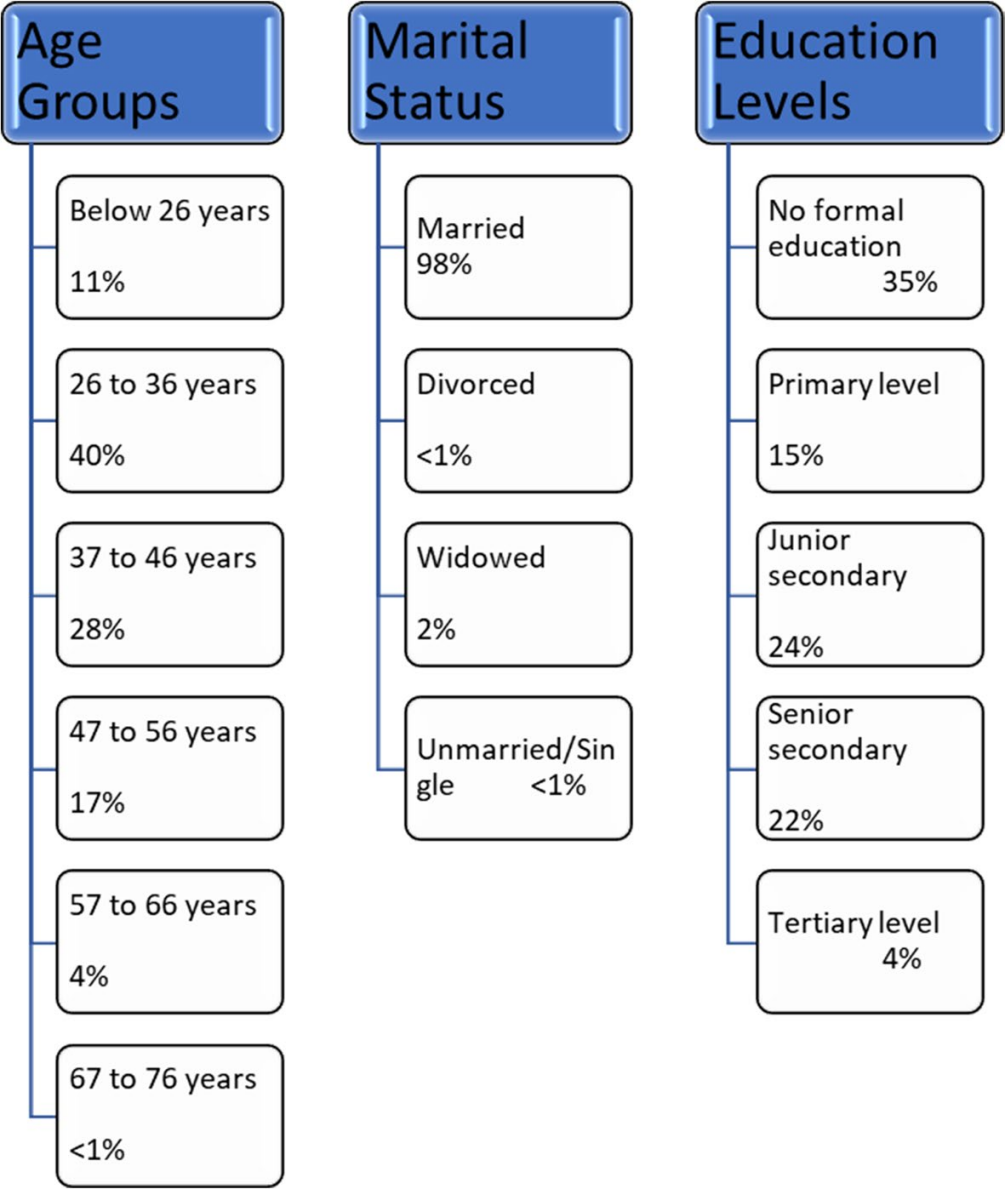
Percentage distribution of individual respondents characteristics

Respondent mean age was 38 years, with the youngest and the oldest being 18 and 75 years respectively (see Fig. 3). Almost all the respondents were married (approximately 2% were divorced, single, or widowed). A majority of the women had some level of formal education, while 35% had no formal schooling. The largest household comprised 14 members and the smallest 2 members (mean household size 4.6 persons). Average monthly household expenditure was 12,398 Bangladeshi taka (BDT) (USD 123.98) in 2020, as at the time of data collection. The mean monthly consumption indicates that households in the study area were living slightly above the international poverty line of $2.15 per day per capita (World Bank 2023).

### 3.2 Results structured around pro-WEFI indicators

#### 3.2.1 Intrinsic agency

Intrinsic agency (power within) is “the process by which one develops a critical. consciousness of one’s own aspirations, capabilities, and rights” (Yount et al. 2019). To evaluate intrinsic agency, we used intrahousehold relationships and autonomy in income indicators. First, to assess adequacy in intrahousehold relationships, 4 statements were posed (Table 2) during the survey. This indicator was analyzed by generating an adequacy score. Women who responded, “high extent” or “medium extent” to each the four questions were allocated a score of “1,” implying that they achieved adequacy in this indicator. Second, to measure autonomy in income, respondents were asked if they were involved in any income-generating activities independently. Those involved in such activities were then asked if they took part in making decisions on how the income was to be used, either individually or jointly with spouses or other household members. Respondents who were involved in such activities, and who solely or jointly participated in making decisions regarding use of the income, were considered to have achieved adequacy in this indicator.

**Table 2:** Descriptive statistics on empowerment indicators

Empowerment domains and indicators	Percentage	Frequency
**1. Intrinsic agency**		
** a. Autonomy in income**		
Women individually involved in income-generating activities.	74%	1223
Range of income earned (BDT)		
Below 2000 (USD 18.54)	82%	1008
2001 (USD 18.5) to 3000 (USD 27.80)	12%	149
3001 (USD 27.81) to 4000 (USD 37.07)	3%	33
4001 (USD 37.08) to 5000 (USD 46.34)	3%	33
Above 5000		
Women involved in income-generating activities and:		
decided how the income was used, solely.	3%	43
decided how the income was used, jointly with husbands.	61%	886
did not participate in deciding use of income.	12%	174
** a. Intrahousehold relationships**		
Percentage of women who had either medium or high extent responses on extent to which:		
They feel their spouse respects them.	98%	1619
They feel adults in your household (in-laws or others) respects them.	99%	1636
They believe their spouse does things that are in respondent’s best interest.	85%	1405
They feel comfortable telling their spouses when they disagree with them.	88%	1453
**2.** **Instrumental agency**		
** a. Access to and control over productive capital**		
Households owned land and pond	100%	1653
Ownership of land and pond		
Land ownership documents solely under husband	68%	1124
Land ownership documents under husband and father-in-law	17%	281
Pond ownership documents solely under husband	59%	975
Pond ownership documents under and father-in-law	19%	314
Land ownership documents under husband and wife jointly	12%	198
Pond ownership documents under husband and wife jointly	12%	198
Land ownership documents under other people in family	2%	33
Pond ownership documents under other people in family	9%	149
Land ownership documents under woman and other people in family	1%	16
Pond ownership documents under woman and other people in family	3%	50
** b. Input in productive and income decisions**		
Women who participated in at least one aquaculture activity	98%	1619
Decision-making in aquaculture-related activities:		
Fish species selection decisions	82%	1355
Brand and type of aquaculture inputs	86%	1421
Fish harvesting related decisions (when to harvest)	82%	1355
Number of fish to be harvested.	88%	1453
Number of fish to be sold.	81%	1339
** c. Control over income **		
Women who participated in money saving decisions.	90%	1488
Women who participated in financial decisions.	80%	1322
Women participated in routine household purchases decisions.	93%	1537
** d. Financial empowerment**		
Respondents household taken any loans or borrowed cash/in-kind from any source in the past 12 months	48%	22%
Respondents has an account at any bank or other formal institutions (e.g. post office)	8%	92%
If yes, please mention about the ownership status of the account	8%	92%
Respondents has an account to digital financial services such as Bkash, Rocket	8%	92%
	Participated	No participation
Woman participated in making decision to take the loan(s) most of the time	74%	16%
Woman participated in decisions about what to do with the money borrowed most of the time	76%	14%
Woman responsible for repaying the loan	41%	58%
Woman participated in making decisions about utilizing the digital account	97%	3%
** e. Mobile phone use and ownership **		
Used mobile (smart/non-smart) phones.	44%	727
Used smart phones	4%	66
Used phone on request (used borrowed phone)	5%	83
** Use of phones **		
Making calls	34%	562
Sending SMS	6%	99
Advertising and marketing	0%	0
Internet-based uses	1%	16
**3. Collective agency**		
** a. Group membership**		
Women with group membership	46%	760
Motivation of joining the group for women with group membership		
Enhancing income	40%	304
Obtaining technical assistance	19%	144
Husband’s suggestion	13%	99
Peer pressure	12%	91
To be part of a network	7%	53
Positive results from group membership as reported by the women who were group members		
Increased technical knowledge	39%	296
Financial benefit	27%	205
Enhanced social status	22%	167
Expanded social network	6%	46

##### Intrahousehold relationships:

Out of the 1653 respondents, 18% had achieved adequacy in intrahousehold relationships (see Table 2). More educated and older women had higher intrahousehold relationship adequacy scores compared to less educated and younger women (see Table 3).

**Table 3:** Statistical tests of relationship between women’s empowerment indicators and women’ marital status, age and education level

Domains of empowerment	Empowerment indicator	Gender characteristics, t-static and significance		
		Marital status Sig. value (std.error/ ms/pr/r/f )	Age Sig. Value (std.error/ ms/r/f)	Education level Sig. value (std.error/ ms/r/f )
Intrinsic agency	**Intrahousehold relationship**	1.4684 (0.267)	4.1958*** (0.65)	12.34*** (0.15)
	**Autonomy in income**			
	Involve in independent income-generating activities	0.0918 (0.762)	−1.6438 (0.578)	17.75*** (0.35)
	Participation in decision to spend independently earned income	0.5908 (0.443)	3.118** (0.93)	5.0247 (3.98)
Instrumental agency	**Input in productive activities and decisions**			
	Number of aquaculture-related activities that woman participated in	2.1858* (0.12)	−0.0685** (0.03)	1.94(7.2)
	Participation in decision-making on productive activities	0.312 (0.67)	−0.2066 (3.54)	5.4627 (5.21)
	**Control over use of income**	1.8361 (1.40)	1.1781 (2.81)	0.41 (8.22)
	**Access to financial services**			
	Ownership of financial account	0.8531 (0.356)	3.659*** (0.75)	46.1*** (0. 00)
	Control of financial account	0.769 (0.09)	−0.0319 (0.97)	0.77 (3.26)
	**Access to and control over productive capital**			
	Ownership of productive resources	−7.809*** (0.005)	0.4721 (0.9)	4.14** (0.01)
Collective agency	**Group membership**			
	Group membership	3.961 (0.151)	−0.2404 (0.511)	13.01** (0.815)
Gender attitudes	Gender attitude	0.716 (0.087)	−0.1089*** (1)	2.5903 (10.01)

Asterisks *, **, and *** represent significance level at 10, 5, and 1% respectively

##### Autonomy in income:

Nearly three-quarters (74%) of the women were independently involved in income-generating activities. These were mainly farm-based (Table 2). The most common activities were livestock and poultry rearing, practiced by 53% and 50% respectively. Entrepreneurship and aquaculture for autonomous income generation were rare. The majority (61%) of the women earned below BTD 2000 (USD 18.41) per month, while 9% earned up to BDT 3000 (USD 27.2) and and 2% earned up to BDT 4000 (USD 36.82), respectively. Only 2% earned BDT 5000 (USD 46.03) or more, while 26% did not engage in any activity (Table 2). Women with higher education levels participated more in independent income-earning activities compared to their non-formally educated counterparts (see Table 3). Most (88%) of the women who individually participated in income-generating activities also participated in making decisions on use of this income. About 12% (143) of the respondents were involved in independent income-generating activities but did not participate in decisions on how to use the income. Instead, these decisions were made by their male spouses (in the case of 90% of women in this category). For a further 10%, their male spouse and father-in-law took the decision together. Test results showed that it was more common for older women in this small band (12% of all respondents) to participate in decisions on use of independently earned income than it was for younger women (see Table 3).

#### 3.2.2 Instrumental agency

Instrumental Agency (or power to) “is strategic action to achieve one’s self-defined goals” (Yount et al. 2019). For this form of agency, input in productive decision-making, women’s involvement in aquaculture activities and gendered distribution and compensation of labor, control over use of income, access to financial services, and access to and control over productive capital were used as indicators.

In assessing women’s input in productive decision-making, respondents were first asked about their participation in various aquaculture-related activities (Table 2) followed by their participation in making decisions on each activity that they took part in aquaculture-related activities including dike excavation, pond preparation, preparation and collection of organic manure, buying of inputs such as feed, fertilizer, and insecticide, buying of fingerlings, stocking of fingerlings, application of fertilizer, feed preparation, feeding, use of lime and other chemicals, disease checking, water quality management, harvesting of fingerlings/fish, selling of fingerlings/fish, marketing of fingerling/fish, marketing, dike cropping and supervision of labor work. They were then asked if they took part in making decisions (Table 2) and the extent of their decision-making (Table 4) regarding fish species selection, brand and type of input, fish time, and quantity of fish to be harvested and sold. Respondents who participated in making the listed decision, either solely or jointly with other people, were considered to have participated in decision-making. Results were presented in descriptive statistics (percentages) (Tables 2 and 4), and test of correlation was conducted in the number of activities that women participated by age, marital status, and the distance of the pond from homestead.

**Table 4:** Gendered distribution of hired and family aquaculture labor

Labor type	Family labor (workdays)		Hired (workdays)		Hired (pay)	
	Male family labor	Female family labor	Male hired labor	Female hired labor	Female labor USD/day	Male labor USD/day
Dike cropping						
Mean	8.56	6.17	3.48	–	–	4.6315
*N*	*210*	*165*	*73*	*–*	*–*	*73*
Guarding						
Mean	109.04	8	118	–	–	2.25
*N*	*24*	*4*	*6*	*–*	*–*	*6*
Harvesting						
Mean	4.92	2.18	5.33	3.67	2.7667	3.2879
*N*	*1438*	*798*	*780*	*3*	*3*	*780*
Marketing						
Mean	3.78	1.45	2.99	5	1.90	2.3874
*N*	*817*	*124*	*261*	*3*	*3*	*261*
Pond preparation						
Mean	3.83	1.4	8.66	26.36	4.2272	4.6194
*N*	*244*	*71*	*93*	*11*	*11*	*93*
Post-stocking management						
Mean	31.95	14.37	9.13	1.5	3.55	4.675
*N*	*1163*	*787*	*16*	*2*	*2*	*16*
Pre stocking management (e.g. liming, fertilization)						
Mean	3.98	1.39	2.55	2.2	3.34	4.4632
*N*	*801*	*241*	*38*	*5*	*5*	*38*
Stocking management						
Mean	6.4	2.17	2.71	2.5	4.10	4.5143
*N*	*1090*	*441*	*14*	*2*	*2*	*14*
Others						
Mean	5.3	1.37				
*N*	*102*	*98*				
Total						
Mean	38.3	9.55	4.22	0.22	4.02	5.13
*N*	*1653*	*1653*	*1653*	*1653*	*23*	*832*

The assessment of women’s involvement in aquaculture activities, and gendered distribution and compensation of labor component, was added in this study to show the participation and engagement of women and men in the aquaculture sector. Participation of men and women in various aquaculture-related tasks through family or hired labor was assessed. In addition to participation, the difference in wage compensation received was used as a proxy to establish whether there was gender inequality in these aspects of aquaculture. In this indicator, descriptive statistics (mean values) were used to analyze data (Table 4).

In assessing women’s control over income use, respondents were asked about their involvement in making decisions regarding saving money, investing in income-generating activities, and routine household purchases of basic items such as food for daily consumption or other household needs, where the household made such decisions. Respondents who had participated in making the decisions either solely or jointly with other people were considered to have achieved adequacy in this indictor.

In this indicator, women were asked if their households had used credit either in cash or kind, in the past one year, and they had participated in deciding to take loan and how the loans were to be used. The respondents were also asked if they owned a financial account, whether a digital one such as Bkash or at any financial institution such as Postbank. Those who had a financial account were then about their involvement in making decisions about utilizing the account and ownership of the account (Table 2). Respondents whose households had accessed loans and participated in making at least one decision about loans, were considered to have an account and had ownership or control, were considered to have achieved adequacy in this indicator.

To evaluate women’s empowerment in access to and control over productive capital an indicator was generated based on owning a pond or land. Respondents were first asked if their households owned land or a fishpond. They were then asked whose name appeared on the ownership document (e.g., land title deed). Women whose households had at least one of the two assets and had their names in the ownership documents, either solely or jointly with other people, were considered to have achieved adequacy in this indicator.

We found that women and men both contributed to family labor in aquaculture. While all surveyed households used labor from male family members in all aquaculture activities, 75% of them used female family labor (Table 4). Across all the activities, female family workdays were lower than male family workdays. For example, the 798 households that utilized female family labor in harvesting recorded an average of 2.18 days, compared to the 1438 that used male labor (an average of 4.92 days).

Women’s participation in fish farming was mostly in feed preparation (72%), feeding (66%) and guarding the pond (47%). About 13% of the female respondents were involved in pond preparation; preparation of organic manure; buying of inputs and fingerlings; stocking of fingerlings; application of fertilizer, chemicals, and lime; dike cropping; and supervision of labor. Around 10% of the women participated in water quality management, pond excavation, disease checking, and selling and marketing of fish and fingerlings. Overall, majority (82%) of the women participated in at least three aquaculture activities.

Age and marital status had a significant relationship with the number of activities that women participated in. The distance of the fishpond from the homestead was significantly correlated with the number of activities that women were involved in (0.0073 p-value, −0.0665 coeff.). In households where fishponds were nearer to the homesteads, women were involved in more aquaculture activities. The average distance of fishponds from the homestead was 0.21 km, with the farthest being 4 km away. While 1% of the households hired female labor, 50% hired male labor. Hired female labor was low across all the activities (Table 3). Notably, female hired labor was not used in dike cropping and guarding, despite woman participating in guarding activities through family labor. A significant difference in compensation for hired labor between men and women was noted across all aquaculture activities.

##### Participation in making aquaculture-related decisions:

Respondents were asked whether they participated in decisions on species selection, brand and type of inputs, fish and fingerling harvesting time, number of fish and fingerlings to be harvested, and the quantity of fish to be sold. Over 80% of the respondents participated in making these decisions (Table 2). A follow-up question was posed, to determine to what extent women participated in decision-making in each of the activities. The responses for involvement were all, some, few, or none of the decisions. As indicated in Table 5, the majority of the respondents were scarcely involved in decision-making under each activity.

**Table 5:** Extent of involvement in decision-making in aquaculture production activities

Activity	Responses in percentages (count/numbers)				
	All decisions	Most decisions	Some decision	Few decisions	No input in decisions
Fish species selection	1 (21)	10 (169)	25 (414)	55 (913)	8 (136)
Brand and type of input	9 (153)	10 (170)	25 (413)	49 (804)	7 (113)
Time of harvesting fish	4 (64)	7 (115)	26 (431)	53 (883)	10 (160)
Amount of fish to be harvested	10 (155)	12 (197)	33 (537)	41 (681)	5 (83)
Amount of fish to be sold	7 (108)	7 (111)	27 (439)	50 (827)	10 (168)

##### Control over use of income:

The majority of the women participated in making income use decisions (Table 2), and 77% had achieved adequacy in this indicator. Over 76% of the women who participated in making the three decisions did so jointly with their husbands, while 5–16% of women reported that income use decisions were made solely by their spouses.

##### Access to financial services:

Results indicate that 4% of the respondents had adequacy in this indicator. Less than half (48%) of the households had used credit either in cash or kind in the past one year. Among the households that had access to credit, 74% of the women had participated in loan taking decisions. In majority of the households (69%), the decision to take and use loans was made jointly by the women and the spouse or together with other household members. In most cases (76%), women participated in making decisions about how to use the loan, though in 52% of the households that had taken loans, the respondents reported that their spouses were in charge of paying the loans.

Women’s access to a digital or an institution-based financial account was very low. The digital financial account example refers to Bkash, Rocket, while an example of institution-based account is any bank such as Postbank. In total, 218 respondents had either a digital or an institutional financial account. About 80% of the women respondents did not have access to any account. Of the 134 (8%) who had digital financial accounts, only 4% had access to a smart phone. The respondent’s age and level of education had a statistically significant association with account ownership. Younger and more educated women were more likely to have financial accounts compared to older and less educated women. In terms of ownership status and control of financial accounts, about 80% of the 134 women had sole ownership of their financial accounts. Decisions on use of the financial accounts was mainly (59%) made jointly by the woman and her husband (Table 2).

##### Access to and control over productive capital:

Findings indicate that all households owned both land and fishponds. About two-thirds (66%) of the women participated in making decisions on what to plant in the household’s land. Ownership documents for the productive capital were mainly under the husband’s name as indicated (Table 2). Overall, 9% (145) of the women achieved adequacy in access and control on this productive asset. The relationship between marital status and adequacy in property ownership was strongly significant. Unmarried women scored higher than married women. Similarly, more educated wome had higher chances of owning productive resources compared to their less educated counterparts.

##### Access to use of mobile phones:

This indicator, though not part of the pro-WEFI, was included for the purpose of this study to add another nuance of the women’s empowerment. In terms of mobile phone use and ownership, it was clear that information communication technology (ICT) penetration among women in the study area was very low. Only 33% of the respondents owned mobile phones, and 88% of them used a non-smart phone. For those who use phones, 35% of the respondents used phones mainly for making calls.

#### 3.2.3 Collective agency

Collective agency (power with) is defined as “joint action to achieve shared goals” (Yount et al. 2019).

##### Group membership:

To assess collective agency, group membership was used as an indicator (Table 2). Women who had membership in groups were considered to have achieved adequacy in this indicator. Aalmost half the women were members of at least one group, implying that they were adequate in this indicator (Table 2). Approximately 5% of the respondents were in group leadership positions. Group membership had a significant association with education level, with more educated women having a higher chance of being members of a group. The most common groups were microcredit (35%), all-women fish farming groups (7%), and mixed-gender farming groups (6%). None of the respondents belonged to a marketing group. The main motivation for joining groups was enhancing income, while the most common positive results from being group members as reported by the women was increased technical knowledge.

##### Gender attitudes:

Gender attitudes were also assessed. To understand the social norms and beliefs that women held, twelve statements based on 5-point Likert scale responses were used to capture respondents’ level of agreement with statements on gender attitudes in aquaculture-related activities. For every positive gender (-equitable) attitude, we allocated a score of “1” and for a non-equitable attitude a score of “0.” For instance, in the statement “women should not get involved in fishing or aquaculture; this is a man’s responsibility”, the responses “disagree” and strongly disagree” were allocated the score “1,” while “strongly agree,” “agree,” and “neither agree nor disagree” were allocated the score “0.” We set the adequacy cutoff as 10 positive scores out of the total of 12 statements.

Results indicate that about 22% of the 1653 women achieved adequacy in gender attitudes. The average gender attitude score was 6 out of 12. In most of the gender attitude statements, about half of the women had positive gender attitudes. Between 50% and 58% of them felt that they, just like men, should be involved in aquaculture and fishing, including activities related to purchasing of inputs and farmer group membership. Approximately 54% of the respondents believed that both genders can earn income from fish trading, can manage a fishpond, and that it is not primarily the man’s responsibility to control income obtained from fish trading. However, more than half the women felt that it was not appropriate for them to get involved in activities such as transporting fish to the market for sale, or to use of boats and nets for harvesting fish. Furthermore, 66% of them were of the opinion that cleaning and cooking fish is the work of women and not men. More details on gender attitudes are in provided in Table 6. The correlation between age and gender attitudes was significant and negative. Older women were more likely to have negative gender attitudes as compared to younger women.

**Table 6:** Assessments of gender attitude statements by the respondents

Gender attitude statements	Strongly agree Percentages and (frequencies)	Agree Percentages and (frequencies)	Neither agree nor disagree Percentages and (frequencies)	Disagree Percentages and (frequencies)	Strongly disagree Percentages and (frequencies)
Women should not get involved in fishing or aquaculture; this is a man’s responsibility	2% (33)	29% (479)	11% (182)	53% (876)	5% (83)
Women should not use fishing nets, boats and other means to harvest fish	4% (66)	52% (859)	10% (165)	31% (512)	3% (50)
Men should not clean, process or cook fish because that work should be done by women	6% (99)	55% (909)	11% (182)	25% (413)	3% (50)
Only men should be able to earn income from trading and marketing of fish	2% (33)	37% (611)	10% (165)	40% (661)	10% (165)
It is not appropriate for women to transport fish to market for sale – only men should do that work	6% (99)	48% (793)	12% (198)	31% (512)	3% (50)
Men should primarily be the ones who control the earnings obtained from the sale of fish	2% (33)	27% (446)	12% (198)	54% (893)	6% (99)
Men should decide on what fish should be brought from the market for consumption	2% (33)	34% (562)	10% (165)	43% (710)	11% (182)
Men should mostly be the ones who belong to fish farmer groups, organizations, cooperatives or associations, not women	2% (33)	28% (463)	11% (182)	56% (926)	3% (50)
Men should make the decisions about buying inputs, selecting feed, and how the pond is managed	3% (50)	41% (678)	12% (198)	41% (678)	3% (50)
Only a man can successfully manage and operate a fishpond.	2% (33)	30% (496)	12% (198)	46% (760)	11% (182)
Trying new ideas in fish farming is for men, not women	2% (33)	39% (645)	14% (231)	41% (678)	4% (66)
The family benefits when men participate in aquaculture training	1% (16)	3% (50)	11% (182)	70% (1157)	16% (264)

## 4. Discussion

### 4.1 Intrinsic agency

#### Intrahousehold relationships

The intrahousehold relationship indicator provides a picture on household harmony (Malapit et al. 2019). The results suggest that household harmony was low, given that only 18% of the women had adequacy for this indicator. Women were least empowered in communicating their disagreement with a particular decision with their spouses. This is the case even though they were not confident that their spouses acted in their (women’s) best interests. This is important because harmonious intrahousehold relationships, and overall strong social cohesion between women and their husbands and other family members, can contribute to other types of empowerment by enabling a woman to do more, including earning income and attending group meetings (Malapit et al. 2019; Meinzen-Dick et al. 2019).

#### Autonomy in income:

Even though the intrahousehold harmony data suggest that in most households’ women did not consider themselves to be in a good position to voice their opinions and/or disagreements, the data on autonomy in income show that in most cases, women had high levels of autonomy in income (74%), meaning they were independently involved in income-generating activities. This shows that lack of harmonious relationships per se did not jeopardize the chances for women to exercise their agency to act and be involved in income-generating activity. Participation in independent income-generating activities provides a good foundation—though it is not the only foundation—for empowerment (Kabeer 1999). Independent income generation is associated with an improvement in women’s status and strengthened women’s decision-making power (Scarborough et al. 2017; Haque et al. 2020; Shirajee 2013; Meinzen-Dick et al. 2019). In this particular case, women were primarilv involved in poultry and small-livestock rearing. These activities are easily managed at homestead level and indeed can be conceptualized as part of women’s reproductive—as much as productive—role because they involve a strong element of care (Anderson and Eswaran 2009; Ahmed and Sen 2018; Quisumbing et al. 2014). This identification appears to be particularly important in rural Bangladesh due to restrictions on women’s mobility (Barman 2001; Jahan et al. 2015; Anderson and Eswaran 2009; Kabir 2013). Such restrictions are associated with maintaining women’s standing in a community (Kabeer 1999) whereas lack of conformity can attract detrimental repercussions such as gossip and loss of respect for the husband (Haque et al. 2020). The mobility of many Muslim women in Bangladesh is constricted by patriarchy and *purdah*^1^ (Mahmud et al. 2012; Shohel et al. 2022). This can have the effect of limiting women’s opportunities to improve their livelihoods and expand their social networks (Parveen and Leonhäuser 2005).

The percentage (61%) of women who participated in decision-making on the use of their independently earned income points to a high level of adequacy in this indicator, among the respondents in the study area. Like our findings, other studies have report fairly high joint decision-making in Bangladesh (Me-Nsope and Larkins 2016; Meinzen-Dick et al. 2019; Kabir 2013). Joint decision-making by women and their husbands can provide a good basis for gender-accommodative and potential gender-transformative initiatives (Haque et al. 2020). Our results additionally corroborate other studies which report that older women and those from single households experience higher participation in income decision-making (Anderson and Eswaran 2009; Kabir 2013; Zaman and Rahman 1999).

A small percentage (12%) of women participated in income-earning activities but were not involved in deciding how income generated from these activities was used. Women may leave decision-making to their spouses and other family members as a sign of respect, or to preserve household harmony. In some cases, they have no desire to make decision solely (Kruijssen et al. 2021).

### 4.2 Instrumental agency

#### Input in productive activities and income decisions

Women’s empowerment in aquaculture requires that they not only participate in aquaculture activities, but this participation should also extend to making related productive decisions (FAO 2017; Ishita 2019). First, our study shows women’s involvement in aquaculture was limited. Gender norms influence the type of activities that women can participate in (Kruijssen et al. 2021). They mainly participated in feed preparation, feeding, and guarding, in that order, and are rarely involved in fish marketing (beyond the farm gate) as would require changes in social norms that limit women’s mobility (Smith and Bhattacharyya 2016; Seymour and Peterman 2018). This conforms with other studies conducted in Bangladesh which report feeding and feed preparation as the major aquaculture activities in which women in Bangladesh are involved (Kruijssen et al. 2021; Haque et al. 2020; Shelly and Costa 2002; Brugere et al. 2001).

Second, our study shows that the majority of respondents participated in making aquaculture-related decisions. Some of these decisions include the selection of fish species for fish farming, selecting the brand and type of input to be used in fish farming, when to harvest fish, the quantity of fish to be sold etc. (Table 2). The most common form of decision-making in aquaculture activities were women jointly with other people in the family. This was different from the pattern observed in making other household decisions where the woman and her husband were the main decision-makers. Sole decision-making by women was rare. Involvement of other family members in aquaculture decisions may be due to the technical nature of fish farming requiring the involvement of other family members for technical advice, or because the pond is considered as an extended family asset.

Women’s participation in the number of fish to sell or to retain for household consumption is important. In Bangladesh, women are typically responsible for securing the family’s nutritional status (Smith and Bhattacharyya 2016).

#### Gendered distribution and compensation, and family and hired labor:

Across all aquaculture activities, family labor provided by women was significantly less than the average workdays provided by men, indicating the dominance of men in aquaculture-related tasks in the study area. Women’s activities in aquaculture are often regarded as supportive of their husband’s input (Kruijssen et al. 2021; Haque et al. 2020; Brugere et al. 2001). As noted above, women’s tasks are primarily associated with feed preparation and feeding. Nevertheless, unmarried and less educated women in the study area were involved in significantly more activities. This indicates a degree of flexibility in gender norms (Meinzen-Dick et al. 2019). In households where ponds were located far from the homesteads, women participated in significantly fewer aquaculture activities, which is in line with other findings on women’s participation in aquaculture (Kruijssen et al. 2021; Brugere et al. 2001).

Hired female labor was low in all the activities. In a study across 16 districts in Bangladesh, Jahan et al. (2015) reported that female family and hired labor employed in *gher*^2^ fish farming technologies was very low compared to male labor. Similar findings were reported by de Brauw et al. (2021) in jute value chains in Bangladesh’s delta region. Possible explanations for limited female family labor in aquaculture could include time constraints due to domestic work, restriction in mobility, religious and cultural norms, and male insecurities (Haque et al. 2020; Balk 1997).

Undervaluation of women’s contribution in aquaculture is further demonstrated by the significantly lower wages for the same activities. Kruijssen et al. (2016) noted that aquaculture benefits in Bangladesh are not equitably distributed between men and women. Similarly, while assessing the barriers and opportunities in agriculture and aquaculture, Haque et al. (2020) reported that women in northern Bangladesh are paid lower wages than men. As further reported by Haque et al. (2020), women are perceived as weak and incapable of working as fast as men, which may partly explain their lower wages. Women may accept low wages due to lack of high-waged alternatives, market supply and demand dynamics (Jahan et al. 2015), and entrenched social norms that attach low value to female labor (de Brauw et al. 2021). Discrimination in hiring and compensating women in aquaculture labor not only compromises their livelihood opportunities but also reduces household income and general well-being (Siddiqua et al. 2021). As noted by Kabeer et al. (2011), participation in paid work enhances women’s power in household decision-making. However, when women are focused into just a few aquaculture activities, this hinders their visibility in aquaculture, hence continued undervaluation of their labor, and under-estimation of their ability.

#### Control over use of income:

More than 80% of the respondents participated in making decisions on the use of household income. These decisions were mainly made jointly by the women and their husbands. Women’s involvement in routine household purchases was higher than all other decisions.

#### Access to financial services:

Aquaculture requires substantial operating capital necessitating access to credit. In the study area, less than half of the households had access to credit. Although a sense of companionship was observed in joint credit-related decision-making, results pointed towards a low adequacy. Access to financial services was very low for women, with over 90% having no access. This was significantly lower among younger women and those who had low levels of formal education. These findings corroborate an Asian Development Bank report by Salman and Nowacka (2020) which noted that women in Bangladesh use insecure and unreliable means of saving money such as clay money saving boxes and buying excess stocks. In bid to improve women’s empowerment through financial services, The Government of Bangladesh and nongovernmental organizations have developed strategies and policies to improve women’s financial inclusion. These include the National Strategy for Social Security, the National Women Development Policy 2011, National Financial Inclusion Strategy (2021–2025) (Asian Development Bank 2022), and “The National Financial Inclusion Strategy” (WFID Partnership 2022). In addition, Bangladesh accounts for over 8% of the world’s mobile money accounts (Rabbani 2020). Despite these facts, data from our study area indicates very low access for women. The gender gap in mobile phone ownership is a significant challenge in women’s financial inclusion (Asian Development Bank 2022). According to Tiwari et al. (2019), in Bangladesh, only 15% and 11% of the women have registered bank accounts and are mobile phone users, respectively.

#### Access to information and extension services:

The data suggest that women’s use of phones to obtain or inquire for information is low: only 29% make calls and 6% send messages. Thus, any effort designed to reach the women with better knowledge, techniques and technology in aquaculture practice need to make use of multiple communication avenues including radio, mobile van in the villages, among others.

There is an opportunity to strengthen inclusivity access to financial services and access to information through multi-sectorial efforts, including ensuring improved use of mobile phones to enhance access to digital financial accounts. This is especially critical with the efforts of the Bangladeshi Government to improve use of ICT in the country through initiatives and policies such as “Digital Bangladesh|:Vision 2020” (Mazumdar and Alharahsheh 2020). The development and growth of e-commerce in Bangladesh has been tremendous over the past two decades. Use of mobile phones and access to financial accounts provides an unexploited opportunity for women in fish farming household to increase their empowerment and participation in the sector through e-commerce (Sultana and Akter 2021). Literature shows that microfinances play a significant role in inclusive financial access and improving women’s empowerment (Islam 2020) and Bangladesh, progress has been made by organizations such as The Grameen Bank of Bangladesh and BRAC (Ali 2021; Islam 2020; Pitt et al. 2006).

#### Access to and control over productive capital

In the study area, almost all the households owned land and ponds. Ownership of productive capital such as land is an indicator of a household’s well-being since these assets are used to generate income and counter shocks (Doss et al. 2014). Gender literature indicates lack of access to, and control over, productive assets is one of the major limitations to empowering women (Meinzen-Dick et al. 2011; Das et al. 2014). Nevertheless, findings from this study indicated that few women had ownership of these assets. Overall, only 9% had adequacy in access and control of ponds and land. This corroborates other studies which report that in Bangladesh, productive assets such as land and ponds are mostly owned by men (Choudhury and McDougall 2018; Jahan et al. 2015; Sproule et al. 2016; Quisumbing and Kumar 2011). Our findings indicate that married women had significantly less adequacy on control over productive capital. According to Meinzen-Dick et al. (2011), women are considered co-owners of their husbands’ assets, but these rights only last as long as they remain married (Kabeer et al. 2011), especially when ownership documents do not include their names. Even when married, women often do not have power to make decision over use of resources without their husbands’ permission (Choudhury and McDougall 2018).

### 4.3 Collective agency

In the pro-WEFI analytic framework, notions of collective agency are indicated by family relationships and group membership.

#### Group membership:

This empowers women through access to inputs and credit, and through social capital and knowledge, among other benefits (Malapit et al. 2019). In addition, group membership interacts with other factors such as financial empowerment and decision-making power, resulting in greater impact on overall empowerment (Gash and Odell 2013). Studies in different contexts across the world demonstrate the importance of collective agency to women’s empowerment (Vimala et al. 2010; Yamashita 2008; Kamal and Rao 2018; Sedai et al. 2021; Gupta et al. 2019; Malapit et al. 2020; Mwambi et al. 2021; Kabeer and Huq 2010). In the study area, almost half the respondents belonged to a group. Given that increasing income was the main reason for joining groups, it is not surprising that micro-credit groups were the most common. Interestingly, increased technical knowledge was the most common benefit.

#### Gender attitudes:

Attitudes to gender roles reflect social definitions of femininity and masculinity, and influence women’s and men’s aspirations and choices. Findings indicate a gap in women’s gender attitudes on aquaculture-related activities. Approximately 22% of women achieved adequacy. Although slightly more than half believed that aquaculture is not just a man’s domain, the majority of women also believed that women should not use fishing nets or transport fish, and that cleaning, cooking and processing fish is not a man’s job. Younger and more educated women had more equitable gender attitudes. Similar findings have been reported in other studies (Balk 1997). The equitable gender attitudes among the younger, educated women may have been as a result of influence from education and exposure to a modern outlook (Sayem and Nury 2013).

Religious, social and cultural norms have a big role inhibiting women’s empowerment, participation, and engagement in aquaculture in Bangladesh (Sultana et al. 2018). For instance, women are discriminated against in asset inheritance (Choudhury and McDougall 2018; Sproule et al. 2016; Kabeer 2011; Lecoutere et al. 2021) and are expected to access land and other productive assets through their male relatives. Practices like benami and naior which encourage women to relinquish their property ownership and control rights to their husbands and brothers, limit asset ownership by women (Quisumbing and Maluccio 2003). Efforts to provide solutions to technical constraints which challenge women’s empowerment, participation, and engagement in aquaculture, need to be accompanied with transformations in critical consciousness (Kantor et al. 2015). Gender Accommodative Approaches (GAAs) are best accompanied by Gender Transformative Approaches (GTAs) to women’s empowerment (Cole et al. 2020). The current study complements existing literature which shows a trend in improved joint decision-making between men and women in Bangladesh (Rahman et al. 2020; Quisumbing et al. 2021). This indicates promising possibilities for GTAs which help to strengthen joint decision-making further. As highlighted by Kantor et al. (2015), effective GTA mechanisms include participatory action research methodologies and behavior change communication strategies.

A self-reinforcing relationship frequently exists between women’s empowerment and economic development. As women take up more opportunities in aquaculture, increased productivity in this sector is observed (Aung et al. 2021; Kruijssen et al. 2018). This contributes to SDGs 1, 2, and 5. As noted by Aung et al. (2021), women’s empowerment in aquaculture also promotes the SDG 14 through increased production efficiency. The findings from this study illuminate a significant opportunity that can be explored to help achieve several sustainable development goals.

## 5. Conclusion

Despite some women achieving adequacy in some indicators, most women in fish farming households in Bangladesh lack adequacy in many of the women’s empowerment indicators assessed. Gaps in adequacy are observed in crucial indicators such as financial empowerment, and access to and control over productive capital. However, joint decision-making was across various activities was reported. Though technical and practical challenges constrain women’s participation and engagement in aquaculture, religious, gender, social norms pose as a big challenge. The authors recommend that aquaculture development projects are target strengthening women’s participation, engagement, and benefit in and from all nodes of the aquaculture value chain, and that women be made visible and valued. In addition, gender-transformative approaches would mitigate the social, cultural, and gender norms that hinder women’s economic, social, and technical empowerment. Special attention should be paid to working with factors such as age, education level and marital status to ensure the benefits of empowerment are experienced widely.

### Limitations of the study

Among the limitations to note in this study is that due to budget constraints, we collected data on seven (7) indicators instead of twelve (12) as required by the pro-WEFI analytic framework. In addition, while in pro-WEFI a final gender parity index (GPI) is calculated from data collected from both women and men from the same household, our study used women-only data, which therefore precluded generating a GPI for more information on WEFI and pro-WEFI (Cole et al. 2020; Ragsdale et al. 2022). We acknowledge that the indicators and patterns reported in this study only point to possible direction of (dis)empowerment and may not paint a conclusive picture of women’s empowerment. Nevertheless, our study provides insights on some of the domains of women’s empowerment in fish farming.

## Data Availability

Data is available upon request.

## Footnotes

1 Seclusion of women from public observation by means of concealing clothing (including the veil).

2 The term *gher* refers to a paddy field which has been modified for shrimp or prawn production. Typically, paddy is cultivated in the middle of the field, which is surrounded by canals with high wide dikes into which the shrimp and prawn are stocked.
